# Sex-dependent effects of mechanical delousing on the skin microbiome of broodstock Atlantic salmon (*Salmo salar *L.)

**DOI:** 10.1038/s41598-023-37670-4

**Published:** 2023-07-04

**Authors:** Elisa Casadei, Amir Mani, Mariela Cisco, Øyvind Vågnes, Irene Salinas, Sonal Patel

**Affiliations:** 1 Department of Biology, Center for Evolutionary and Theoretical Immunology, University of New Mexico, Albuquerque, NM 87131 USA; 2 Vaxxinova Norway, Kong Christian Frederiks Plass 3, 5006 Bergen, Norway; 3 Blue Analytics AS, Kong Christian Frederiks Plass 3, 5006 Bergen, Norway; 4 Norwegian Veterinary Institute, Thormøhlens Gate 53C, 5006 Bergen, Norway

**Keywords:** Molecular biology, Bioinformatics, Microbial communities

## Abstract

Delousing strategies, including mechanical delousing, are typically used to treat Atlantic salmon (*Salmo salar)* sea lice infestations. In this study, we evaluate the impact of mechanical delousing (Hydrolicer) on the skin bacterial microbiome of broodstock female and male Atlantic salmon. 16S rDNA sequencing of salmon skin microbial communities was performed immediately before delousing, right after delousing and 2 and 13 days post-delousing (dpd). The skin bacterial community of female salmon was more diverse than that of males at the start of the experiment. Overall, hydrolycer caused losses in alpha diversity in females and increases in alpha diversity in males. Hydrolicer also caused rapid shifts in the skin microbial community composition immediately after delicing in a sex-specific manner. There was a decrease in abundance of Proteobacteria and Bacteriodetes in both female and male salmon, whereas Firmicutes and Tenericutes abundances increased. Interestingly, the female community recovered faster, while the male community remained dysbiotic 13 dpd due to expansions in Bacteroidetes (*Pseudomonadaceae*) and Firmicutes. Our data suggest that female broodstock are more resilient to Hydrolicer treatment due to their more diverse skin microbiota community, and that sex influences the skin microbial community and therefore host health outcomes during common farming manipulations.

## Introduction

Microbiota composition is governed by variables such as environmental factors, host genetics, development, diet and sex^1–4^. Host biological sex is a determinant factor of human skin and gut microbial community composition^5^. However, studies on several other vertebrates found no evidence for biological sex to be a significant variable shaping skin microbial communities^6–8^^.^ Recent studies have explored the relationship between fish biological sex and skin microbiome^9–11^. For instance, female guppies found to have a higher abundance of certain bacterial taxa, such as Flavobacterium, compared to males^9^. These differences are thought to be driven by differences in hormonal profiles and immune function between the sexes. Sex may also play a role in how microbiota shifts in response to perturbations, a complex interaction due to the effect of sex hormones on the adrenal axis.

Microbial ecology theory defines resilience of a microbial community as the ability of the community to restore its equilibrium following a perturbation^12^. Resilience is determined by the capacity to reduce the impact (resistance) and to recover from the impact of disturbance (recovery)^13^. Overall, more diverse microbial communities are thought to be more resilient^14^. Resilience of microbial communities may also be shaped by host biological sex. For example, mice exposed to different stressors early in life showed alternations in the gut microbiome that were sex-dependent^15^. In fish, several perturbations are known to impact microbial community composition including diet, infection, toxicant exposure, antibiotic administration, stress, and others^16–19^. However, the effects of biological sex on fish microbiota and how biological sex impacts the effects of perturbations on the fish microbiome remain largely unexplored.

Biological sex not only impacts microbiota but also the type and magnitude of physiological responses to stress. One of the most widely reported findings is that female rodents have higher levels of HPA axis hormones than males^20^. Specifically, basal levels of the glucocorticoid, corticosterone, are higher in females compared to male rats^20^. In response to stress, human studies have reported a greater cortisol response in males than females^21^. In fish, sockeye salmon males and females differ in cortisol responses to stress^22^. Specifically, cortisol levels are higher in females than in males during their spawning period, and females might be less sensitive to changes in the level of cortisol caused by acute stress compared to males.

The ectoparasite *Lepeophtheirus salmonis* or sea lice is a major infectious agent in farmed and wild salmonids in Norway^23,24^. *L. salmonis* infests Atlantic salmon at all stages of salmon life in the seawater phase^25^ with varying parasitic loads depending on parameters such as host density, and seasonal variations. Salmon broodstock kept in open sea cages are just as likely to be infested with *L. salmonis* as the salmon being produced for food consumption. Sea lice infested salmon undergo several physiological responses such as cortisol production due to stress response and osmoregulatory changes^26^. Furthermore, the impact of sea lice infestation on the skin microbiome of salmon was recently assessed^27^. These studies determined that *L. salmonis* infestation results in loss of microbial diversity and that infested fish with high parasite burdens, harbor multiple pathogenic bacterial taxa including *Vibrio, Flavobacterium, Tenacibaculum,* and *Pseudomonas*^27^. Furthermore, *L. salmonis* can act as a vector for fish pathogens such as *A. salmonicida* and IHNV^28^. Combined, this body of work highlights the complex interactions between host, parasitisation, stress and microbiota.

Atlantic salmon farming at high density exacerbates the negative impact that *L. salmonis* has on both farmed and wild fish. In some countries, this impact is regulated by a legislative threshold of lice dictating when delousing treatments must occur. In Norway, this level is 0.2 adult female lice per fish during the spring salmon migration period and 0.5 the rest of the year^23^. Thus, every year, farmed Atlantic salmon are subject to several delousing treatments to remove the lice attached on salmon. These treatments may consist of one or more different delousing methods including chemical, biological, thermal and mechanical treatments. Due to increased resistance to chemical treatments, thermal and mechanical treatments are currently the preferred methods of choice^29^. However, these thermal and mechanical treatments are associated with increased mortality that may be derived from the stress associated with the delousing handling and crowding as well as physical harm to the fish^29^. For instance, mechanical delousing involves the use of a high-pressure tumble that prompts the sea lice detachment from the salmon surface. Furthermore, mechanical treatment may also damage external mucosal barriers and their associated microbial communities, a perturbation that may facilitate opportunistic bacteria colonization. The goal of the present study is to evaluate (1) whether mechanical delousing using Hydrolicer treatment has an impact on the skin microbial community of Atlantic salmon broodstock and (2) whether biological sex is a determining factor in the dynamics of microbiome recovery following delousing. Our findings indicate that the skin microbiome of Atlantic salmon broodstock shifts quickly in response to mechanical delousing and that host biological sex is an important factor determining the response of the microbiome to this perturbation.

## Results

**Figure 1:**
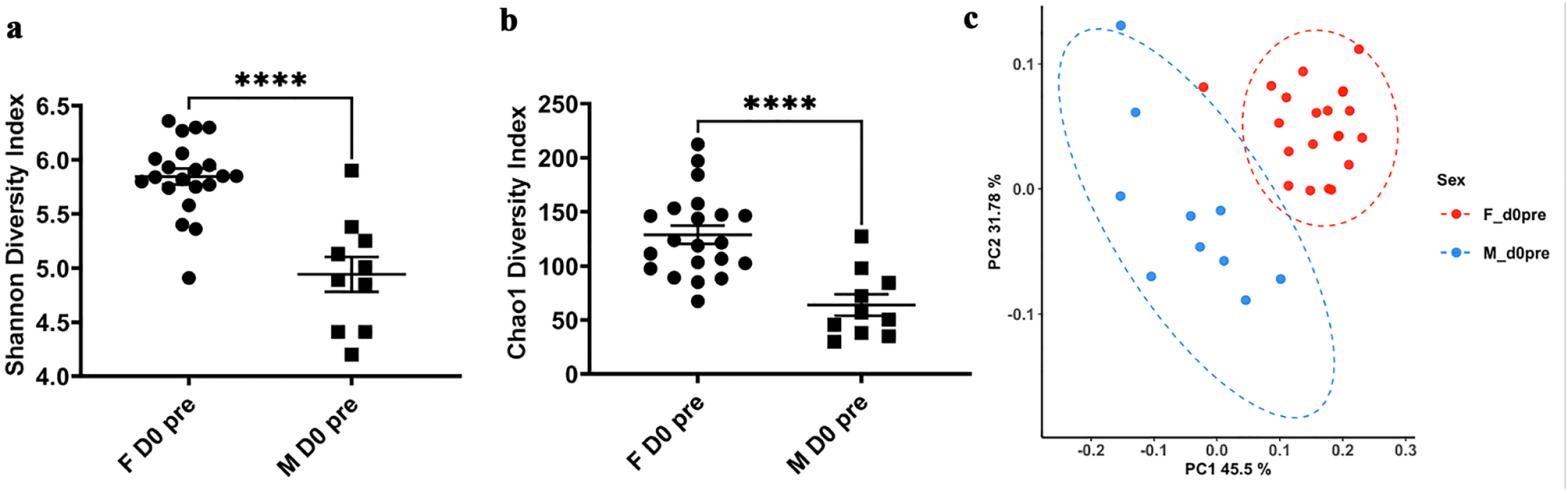
Sex differences in broodstock Atlantic salmon skin microbial communities. (**a**) Mean Shannon diversity index of female and male Atlantic salmon skin microbial communities at time 0 pre-delousing (start of the experiment). (**b**) Mean Chao 1 index of female and male Atlantic salmon skin microbial communities at time 0 pre-delousing (start of the experiment). (**c**) Principal coordinate analysis of female and male broodstock Atlantic salmon skin microbial communities at time 0 pre- delousing. **** indicates a *P-*value < 0.0001 by t-test. Ellipses show significance at a confidence interval of 95% (*P* < 0.05)^30^.

16S rDNA sequencing of skin samples prior to delousing indicates that male and female salmon had very different microbial communities. Specifically, the mean alpha diversity (Shannon diversity index and Chao 1) of the female salmon skin microbiome was significantly higher than that of male salmon (Fig. 1a–b). Principal coordinate analyses of the female and male microbial communities showed distinctive grouping based on sex, with the female samples showing a tighter distribution indicating less heterogeneity compared to males (Fig. 1c).

**Figure 2:**
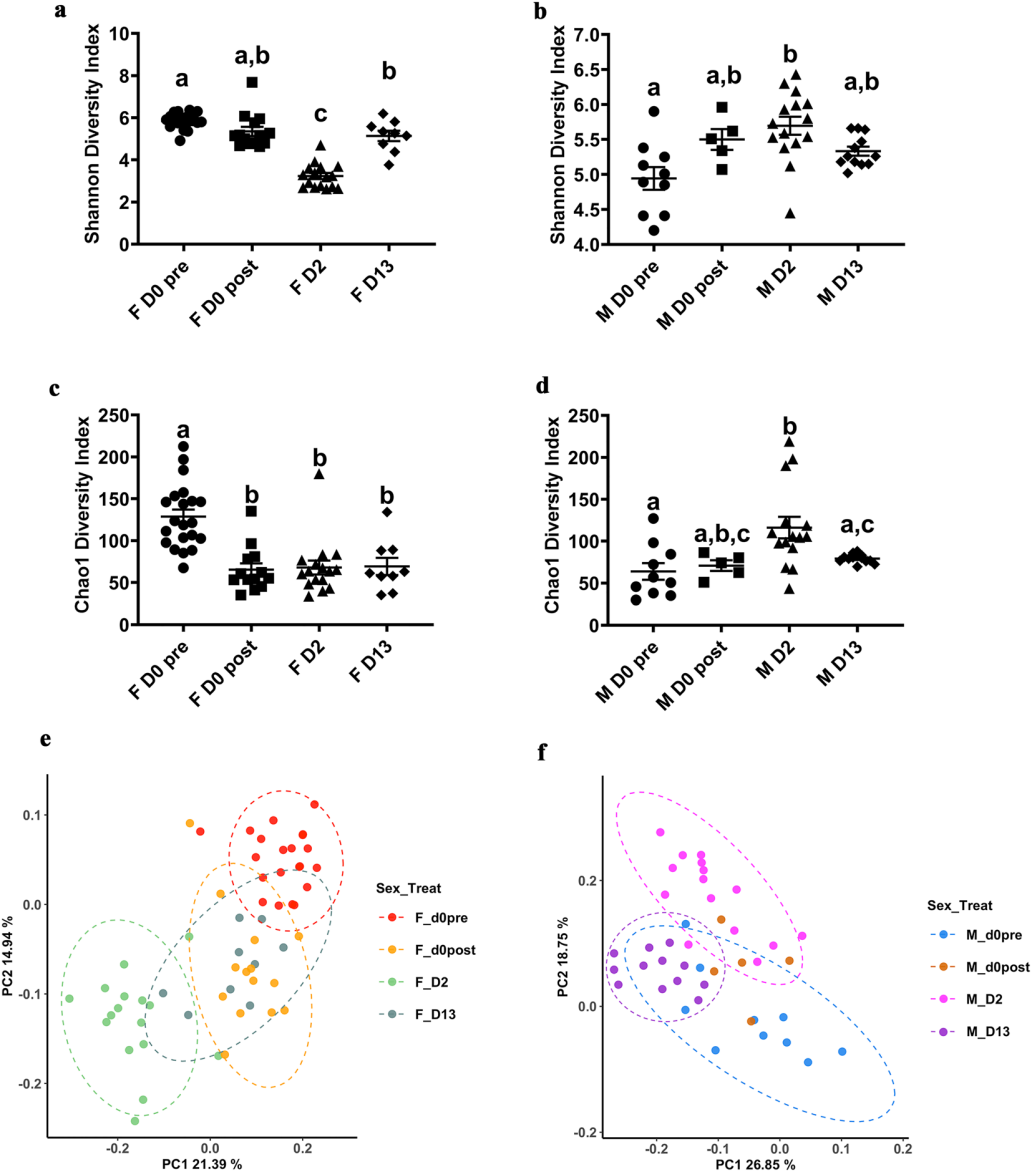
Effects of mechanical delousing on the skin microbial community of female and male broodstock Atlantic salmon*.* Mean Shannon diversity index of female (**a**) Atlantic salmon and (**b**) male Atlantic salmon skin microbial communities at day 0 pre-delousing, day 0 post-delousing, 2 dpd and 13 dpd. Mean Chao-1 index of female (**c**) Atlantic salmon and (**d**) male Atlantic salmon skin microbial communities at day 0 pre-delousing, day 0 post-delousing, 2 dpd and 14 dpd. Principal component analysis of female (**e**) Atlantic salmon and (**f**) male Atlantic salmon skin microbial communities at day 0 pre-delousing, day 0 post-delousing, 2 dpd, and 13 dpd. Different letters designate statistically significant groups using one-way ANOVA followed by Sidak’s test for multiple comparisons with a *P *< 0.05. Ellipses show significance at a confidence interval of 95% (*P* < 0.05)^30^.

Following delousing, we observed immediate losses at 0 days post delousing (dpd) in alpha diversity (Chao1) in the skin microbial community of females but not males (Fig. 2a–d). The mean Shannon diversity index in the female salmon skin microbial community started to decrease right after delousing and reached its lowest mean value at 2 dpd. By day 13, the Shannon diversity index of the female skin community was still significantly lower than that at the day 0 pre-delousing controls but higher than that at 2 dpd (Fig. 2a).

In females, mechanical delousing also resulted in decreases in the mean Chao 1 values, which remained significantly lower during the entire duration of the sampling period compared to day 0 pre-delousing controls (Fig. 2c).

In males, shifts in the skin microbiome alpha diversity following delousing were very different than in females, and characterized by increases rather than decreases in both Shannon and Chao1 alpha diversity values which was statistically significant only at 2 dpd compared to pre-delousing group (Fig. 2b and d). The mean Shannon diversity index of the male community started to increase immediately after delousing and reached its highest value at 2 dpd. By day 13 the mean Shannon diversity index of the male skin community, although not any more significant, was still elevated compared to pre-delousing controls, but it was lower than at 2 dpd (Fig. 2b). Chao 1 values showed a similar trend, reaching their highest value 2 dpd and returning to basal levels by day 13 (Fig. 2d). PcoA plots indicate that in both females and males, delousing at 2 dpd, caused the greatest differences in the skin microbial communities (Fig. 2e–f).

Mixed model ANOVA analyses indicate that sex, treatment (time post-delousing) and the interaction between sex and treatment are all significant factors in the alpha diversity of the Atlantic salmon skin microbial community (Table 1).

**Table 1:** Mixed model ANOVA analysis.

Source	F value	*P* value
Sex	82.63	2.37e^−15^***
Treatment (Time)	47.73	2e^−16^***
Sex: Treatment	68.49	2e^−16^***

Combined, these results indicate that the alpha diversity of the skin microbial community of Atlantic salmon is significantly altered by mechanical delousing and that sex plays a significant role in the response with females losing diversity and males increasing diversity as a response to delousing.

**Figure 3:**
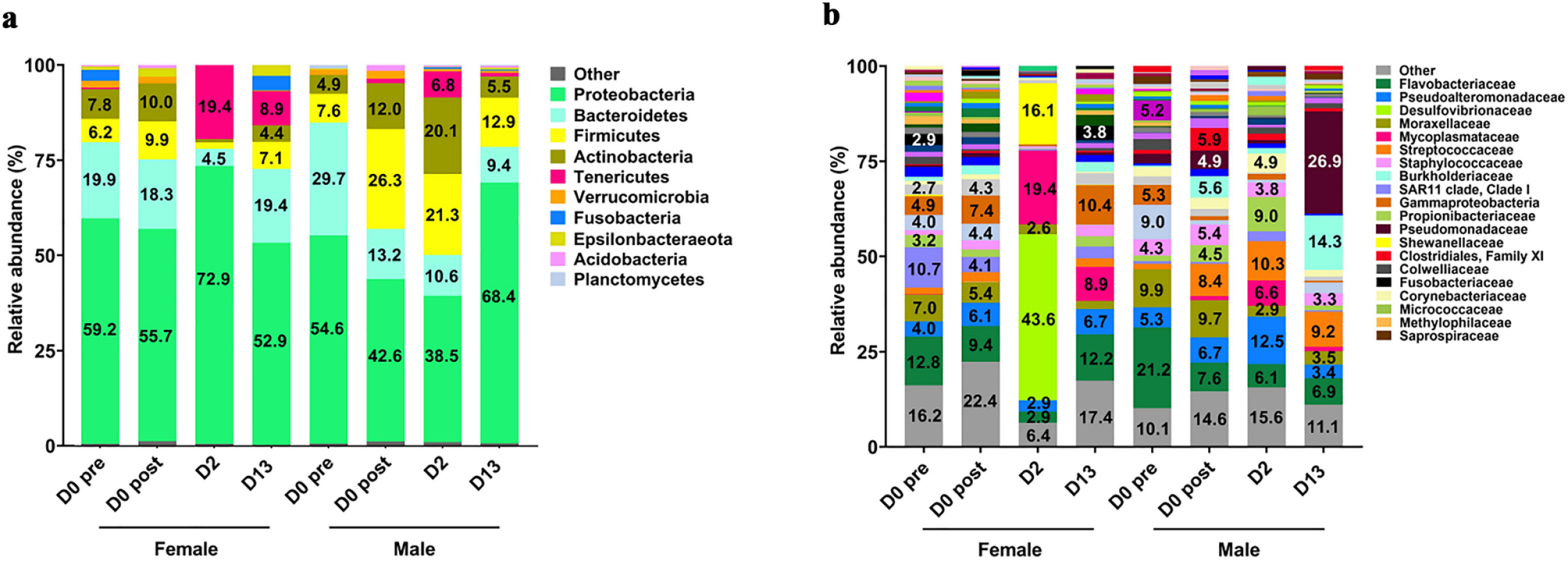
Delousing changes the bacterial community composition of the Atlantic salmon skin microbiome. (**a**) Relative abundance at the phylum level of the female and male skin microbial community composition at day 0 pre-delousing, day 0 post-delousing, 2 dpd and 13 dpd. (**b**) Relative abundance at the family level of the female (**a**) and male (**b**) skin microbial community composition at day 0 pre-delousing, day 0 post-delousing, 2 dpd and 13 dpd.

At the phylum level, Proteobacteria dominated the skin microbial community of both females and males at all sampling points. Prior to delousing, Proteobacteria accounted for 59.2% of the total community in females, whereas in males, Proteobacteria accounted for 54.6% of the overall diversity. Interestingly, Bacteroidetes abundance was higher in males than females (29.7% and 19.9%, respectively), while the abundance of Firmicutes was similar in females and males (6.2% and 7.6%, respectively) prior to delousing (Fig. 3a).

Mechanical delousing caused rapid shifts in the skin bacterial community composition of Atlantic salmon broodstock. Specifically, on day 0 post-delousing, the contribution of Proteobacteria to the skin microbial community in males decreased from 54.6% to 42.6% whereas in females Proteobacteria abundance only dropped from 59.2% to 55.7%. Bacteroidetes abundance drastically dropped from 29.7% (day 0 pre-delousing) to 13.2% (day 0 post-delousing) in males. The abundance of Firmicutes, in turn, increased from 7.6% (day 0 pre-delousing) to 26.3% (day 0 post-delousing) in males. In females, changes in Bacteroidetes and Firmicutes abundances right after delousing were very minor (Fig. 3a).

At 2 dpd, females showed the largest shift in microbial community composition with a significant expansion in Proteobacteria abundance (72.9%) compared to pre-delousing levels (59.2%) as well as dramatic expansion in Tenericutes abundance (19.4%) compared to pre-delousing levels (< 1%) (Fig. 3a). The abundance of Tenericutes was 8.9% in the female skin microbial community at 13 dpd. The abundance of all other phyla was similar at day 13 compared to day 0 pre-delousing.

Changes over time in males were very different from females. Delousing resulted in losses of Proteobacteria abundance that started right after delousing and persisted by day 2. By day 13, Proteobacteria abundance had bounced back and was significantly higher (68.4%) than in males at time 0 pre-delousing (54.6%). Delousing caused losses in Bacteroidetes abundance in the male skin microbial community that persisted throughout the duration of the sampling, going from 29.7% pre-delousing to 13.2% right after delousing, to 10.6% at 2 dpd and 9.4% at 13 dpd. Thus, in males, compared to females, Bacteroidetes abundance did not recover to basal levels (Fig. 3a).

Interestingly, dramatic changes in the relative abundance of Firmicutes were observed in males increasing from 7.6% on day 0 pre-delousing to 26.3% right after delousing, 21.3% 2 dpd and 12.9% 13 dpd. As mentioned earlier, this contrasts with changes in Firmicutes in females which only significant decreased at 2 dpd (0.63%) compared to pre-delousing levels (6.2%) (Fig. 3a).

**Figure 4:**
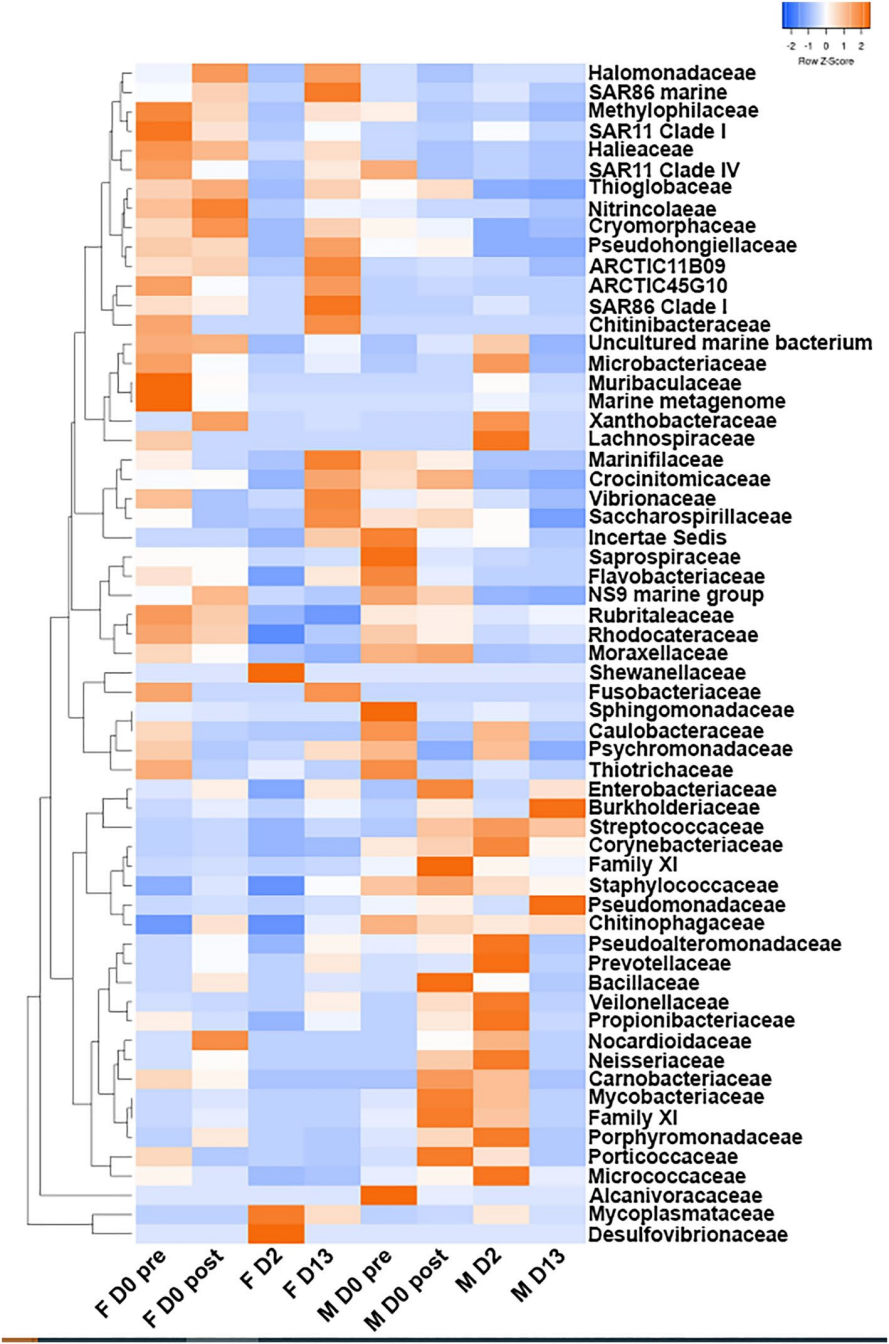
Significantly different ASVs in female and male Atlantic salmon skin microbial community day 0 post-delousing, 2 dpd and 13 dpd.

**Figure 5:**
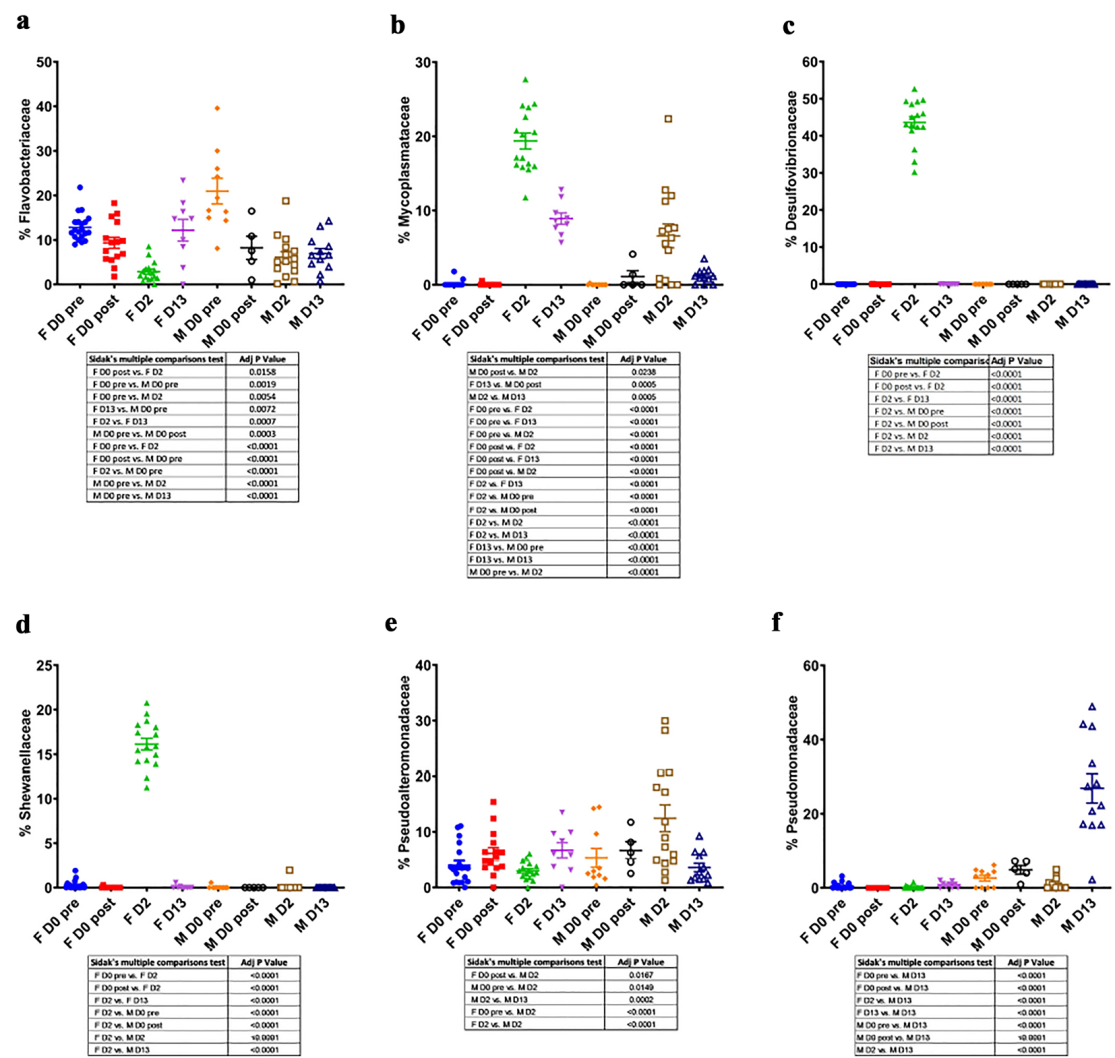
Delousing causes differential expansions of bacterial taxa in female and male skin microbial communities. Relative abundance of (**a**) *Flavobacteriaceae*, (**b**) *Mycoplasmataceae*, (**c**) *Desulfovibrionaceae*, (**d**) *Shewanellaceae*, (**e**) *Pseudoalteromonadaceae* (**f**) *Pseudomonadaceae* in female and male Atlantic salmon skin microbial communities at day 0 pre-delousing, day 0 post-delousing, 2 dpd and 13 dpd. *P-*values for Sidak’s multiple comparisons test are shown in the tables underneath each of the graphs.

The microbial community composition at the Family level revealed striking differences in the effects of delousing on female and male salmon (Figs. 3b, 4 and 5). As previously mentioned, female skin microbial communities had the lowest alpha diversity values at 2 dpd. This was due to the changes in abundance of three families, *Desulfovibrionaceae* which accounted for 43.6% of all diversity, *Mycoplasmataceae* (which accounted for 19.4%) and *Shewanacellaceae* (which accounted for 16.1%) compared to almost negligible levels at day 0 pre- and post-delousing (Figs. 3b, 4 and 5b–d). Interestingly, by day 13, the female skin microbial community composition had lost *Desulfovibrionaceae* but *Mycoplasmataceae* still accounted for 8.9% of the overall diversity. *Flavobacteriaceae* abundance, which accounted for 12.8% of the overall community at day 0 pre-delousing in females and dropped to 2.9% at 2 dpd, recovered at 13 dpd with a similar abundance (12.2%) to that of pre-delousing controls (Figs. 3b, 4 and 5a).

In males, *Flavobacteriaceae* abundance dropped right after delousing accounting for 7.6% of the overall abundance on day 0 post-delousing compared to 21.2% on day 0 pre-delousing.

*Flavobacteriaceae* abundance stayed at significantly lower levels in males throughout the sampling period (Figs. 3b, 4 and 5a). Interestingly, the family *Streptococcaceae* which contains potentially pathogenic bacterial taxa, was expanded in males right after delousing (8.4% compared to 1.4% pre-delousing) and stayed elevated for the remaining period (10.3% and 9.2% at 2 and 13 dpd, respectively) (Figs. 3b, 4 and Supplementary Fig. 1b and supplementary table 1). At day 2, the skin microbial community in males showed an expansion in the abundance of the family *Pseudoalteromonadaceae (*Figs. 3b, 4 and 5e). By day 13, at the family level, the male microbial community composition was highly dissimilar to that of pre-deloused male salmon. This was largely due to the high abundance of *Pseudomonadaceae* (26.9%) which was previously < 5% at all the other sampling points (Figs. 3b and 5f). Additionally, *Burkholderiaceae*, found at very low abundance in all other male groups, accounted for 14.3% of the overall diversity at 13 dpd (Fig. 3b, 4 and Supplementary Fig. 1a). We also noted increased abundances of *Moraxellaceae* (Supplementary Fig. 1c), *Propionibacteriaceae* (Supplementary Fig. 1e) and *Mycobacteriaceae* (Supplementary Fig. 1f.) in males at different time points post-delousing. Finally, we observed significant losses in the abundance of SAR11 in females in all deloused female treatment groups (Supplementary Fig. 1d).

These results indicate that the increased diversity of the male Atlantic salmon skin microbial community is the result of colonization by potentially opportunistic and pathogenic taxa.

**Figure 6:**
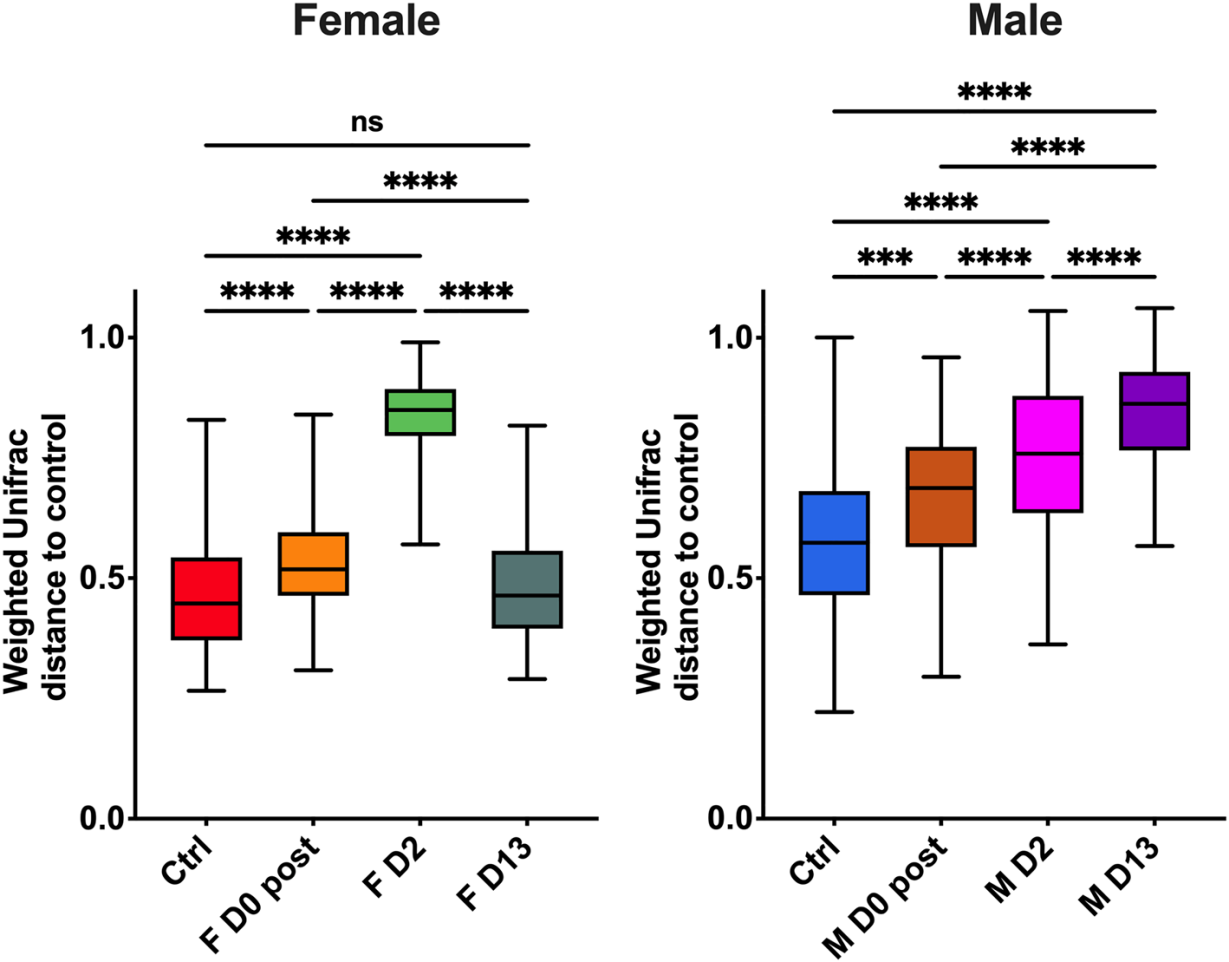
The skin microbial community of female Atlantic salmon recovers quicker from delousing perturbance compared to males. Weighted UniFrac distance of the female skin microbial community compared to day 0 pre-delousing in females (**a**) and males (**b**). ***indicates a *P*-value < 0.001 and ****indicates a *P*-value < 0.0001 by t-test.

In order to establish the ability of each of the pre-disturbed communities to recover from the delousing perturbance, we analyzed the Weighted UniFrac distances of each of the female and male microbial communities compared to pre-deloused controls. As shown in Fig. 6, the female skin microbial community was most dissimilar to the pre-deloused control at day 2, but by day 13, the community was no longer different from that of day 0 pre-deloused salmon.

This was in sharp contrast with the kinetics observed in males, where the Weighted UniFrac distance of the community between pre-delousing and post-delousing treatments became greater over time, indicating no recovery post-perturbance and a lower resilience of the skin microbial community compared to females.

**Figure 7:**
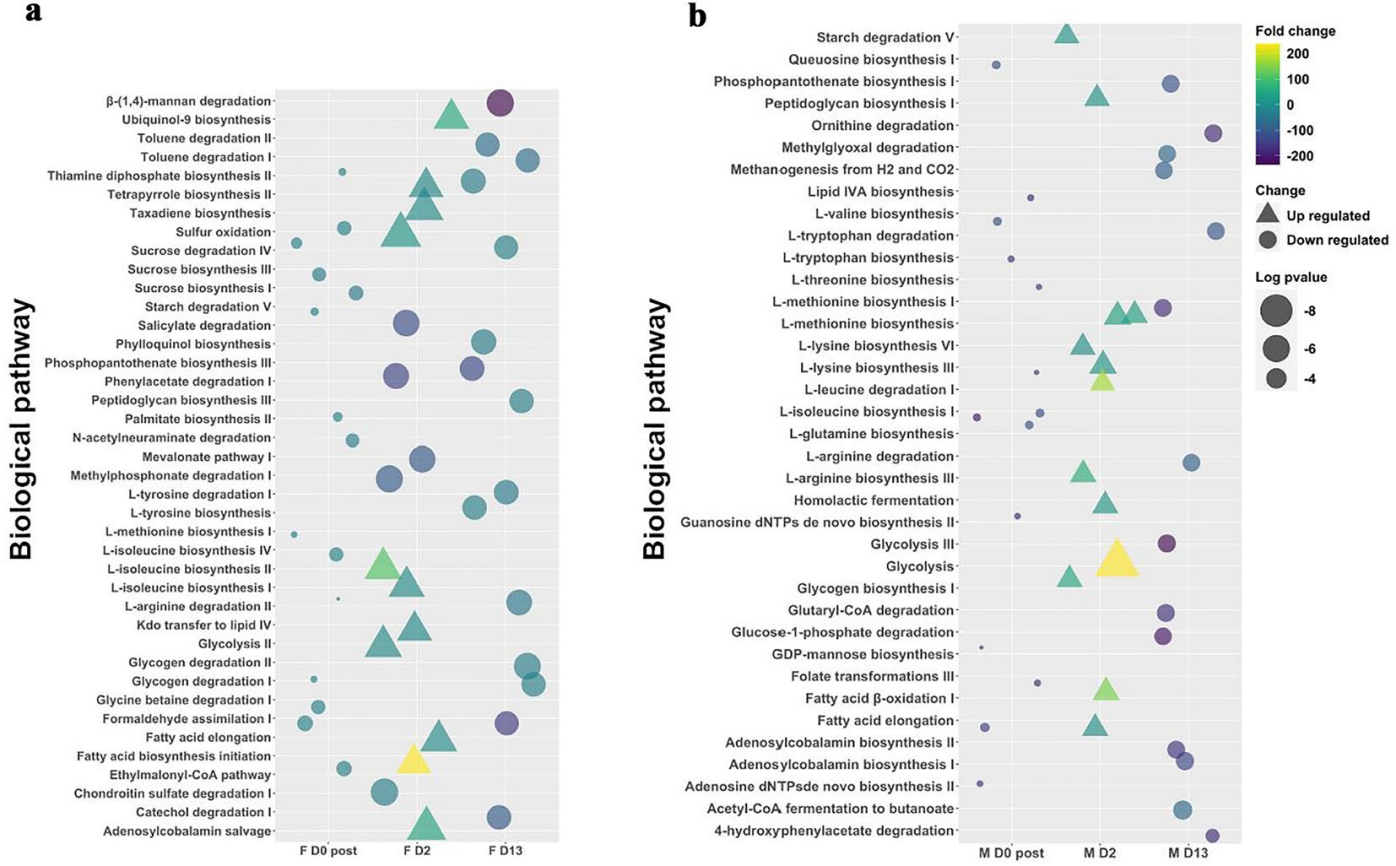
PICRUSt2 analysis of the skin microbial communities of female and male Atlantic salmon following delousing predicts sex-dependent altered biological pathways. (**a**) Predicted biological pathways significantly altered in the female Atlantic salmon skin microbial community day 0 post-delousing, 2 dpd and 13 dpd compared to day 0 pre-delousing. (**b**) Predicted biological pathways significantly altered in the male Atlantic salmon skin microbial community day 0 post-delousing, 2 dpd and 13 dpd compared to day 0 pre-delousing. For each sex only the top 10% of the significantly modified pathways are shown.

We next performed PICRUSt2 analysis to determine what biological pathways in the skin microbiota may be impacted by mechanical delousing perturbance. Of note, these pathways may change not only to the stress associated with the Hydrolicer treatment but also in response to the removal of sea lice from the salmon’s external barriers. We identified a total of 114 significantly up-regulated pathways and 271 significantly down-regulated pathways in deloused female Atlantic salmon compared to day 0 pre-delousing controls. Most of the significantly modified pathways were detected at 2 dpd in both females and males with very few pathways detected at day 0 post-delousing (Fig. 7). Interestingly, in general, we noted an overall upregulation in biological pathways at day 2 and an overall downregulation of pathways at day 13 in both sexes. The most up-regulated pathway in females was β-(1–4) mannan degradation at 2 dpd (Fig. 7a). Other upregulated pathways at day 2 included isoleucine biosynthesis, ubiquinol-9 biosynthesis, taxa diene biosynthesis, sulfur oxidation, glycolysis II, fatty acid elongation and the adenosylcobalamin salvage pathway. By day 13, predicted down-regulated pathways included the β-(1–4) mannan degradation pathway, toluene degradation, sucrose degradation, glycogen degradation and catechol degradation.

In males, again, minor changes in biological pathways were predicted right after delousing, most of the significantly different pathways occurring at day 2 and some at day 13. At day 2, the most up-regulated pathway was glycolysis, followed by fatty acid β-oxidation and many amino acid biosynthesis pathways such as L-arginine biosynthesis, L-lysine biosynthesis, L-methionine biosynthesis and L-leucine biosynthesis. Starch degradation and the peptidoglycan synthesis pathway were also upregulated at 2 dpd. By 13 dpd, Glycolysis III, glutaryl-CoA degradation and glucose-1 phosphate degradation pathways were all significantly down-regulated. The tryptophan and arginine degradation pathways were also downregulated at this point as well as the ornithine degradation pathway. Combined, these results suggest that the skin microbial community of female and male Atlantic salmon undergoes very different changes in carbon, fatty acid and amino acid metabolism following mechanical delousing perturbance.

## Discussion

Fish farming is associated with man-made stressors due to handling, sorting, transport, and vaccination of fish among others. In the case of Atlantic salmon, the constant infestations caused by sea lice have resulted in routine delousing practices to reduce parasitic load in the farmed salmon and therefore minimize impact on wild fish populations. Mechanical delousing is inherently associated with fish handling and stress, which in turn may render salmon more susceptible to secondary infections and cause mortalities.

Fish mucosal barriers have diverse bacterial communities living in symbiosis with the host^31,32^. Fish microbiota quickly respond to any host-derived and environmental-derived perturbations. In our study, changes in the salmon skin microbiome in response to mechanical delousing may therefore be the result of direct and indirect mechanisms. Mechanical delousing can directly impact the microbial community composition of the salmon skin. Few reports have considered the effect of mechanical delousing on fish health and wellbeing reporting scale loss, skin damage and gill hemorrhages^29,33^. Moreover, delousing can induce a stress response on the salmon, which, in turn, will impact microbial community composition^16^. In addition to these layers of complexity, we added one more variable, which is biological sex. This comparison suggests unique responses of female and male broodstock salmon in skin microbiomes to mechanical delousing.

We found that the skin microbial community of female broodstock salmon was much more diverse than that of male salmon. Previous studies in mice also found that male mice had lower microbial diversity in the gut microbiome compared to females^34^ whereas in humans, conflicting results exist depending on the study^35^. The females and males were from same batch of eggs, were maintained in same tank at hatchery, had an identical vaccination history and were differentiated just before transfer to sea. Post seawater transfer, they received the same diets, and there was no differences in infection or disease situation between the cages, and thus we predict that the sex effects detected in the skin microbiome are likely due to the effects of sex hormones, metabolism and/or body mass index^35^. Finally, while our sample size was large and provided us with strong statistical power, we, however, had no replicate cages in our study and therefore we need to be cautious about a cage-effect in our dataset. Because female and male animals used in our study were sampled from open sea cages in near proximity, the water microbial community should have been identical in both cages.

Resilience is the amount of stress or perturbation that a system can tolerate before its trajectory changes towards a different equilibrium state^36^. Currently, baseline microbiome analyses prior to any manipulation are not performed in fish farms due to the economic costs associated with such analyses. Our results suggest that the female salmon skin microbial community is more resilient to the perturbation caused by delousing stress. This finding agrees with the current theory that more diverse microbial communities are more resilient to perturbations because of functional redundancy among taxa^37–39^. Nevertheless, as cage and sex effects cannot be separated in our study, further research using replicated single sex populations or sampling of males and females from mixed-sex cages is warranted. We also noted opposite trends with regards to Chao 1 and Shannon alpha diversity changes in the female compared to male skin microbial community. Specifically, we noted that the female community became less diverse in response to delousing, suggesting losses in some taxa that may become dislodge upon the mechanical stress and/or detachment of parasites. In males, however, delousing caused increases in alpha diversity in the skin microbial community, indicating colonization of new taxa upon delousing. Combined, our results highlight the value of microbiome health prior to stressful manipulations in salmonid farming with the caveat that our studies should be extended to other life stage such as grow-out fish to determine if broodstock and grow-out animals show similar trends.

The post-disturbance equilibrium of microbial communities is often resilient since these taxa may be already resistant to the same perturbance^40^. Our data indicate that female and male Atlantic salmon skin microbial communities were different at 13 dpd and that the male community maintained a distinct composition compared to the day 0 pre-deloused community as time went by. These results show distinct skin microbial community responses in female versus male Atlantic salmon following mechanical delousing and suggest that male salmon skin microbial communities take longer to recover compared to female salmon. Female salmon, on the other hand, appear more susceptible to colonization by *Mycoplasmataceae*, a taxon typically found in the gut but not the skin microbial community of salmonids^10^, following delousing perturbation. Atlantic salmon are deloused many times during their life depending on *L. salmonis* infestation levels. Thus, future studies may evaluate how post-disturbance communities respond to repeated delousing and highlight differences in male and female microbiota responses to cycling delousing perturbances.

Atlantic salmon are susceptible to several bacterial pathogens following delousing^41–43^. Currently, problematic skin pathogens that affected farmed Atlantic salmon in Norway include *Moritella sp.*, and *Tenacibaculum sp*.^44,45^ and co-infections with *M. viscosa* and *L. salmonis* have been reported^46^. Interestingly, we did not detect any ASVs corresponding to these problematic bacterial taxa in our dataset. In agreement, previous studies failed to detect *Vibrio sp.*, *Flavobacterium sp.*, *Tenacibaculum sp*., or *Pseudomonas sp*. on an individual level, but network analysis of microbial taxa on lice infested fish revealed the association of multiple pathogenic genera with high louse burdens^27^. At the genus level, due to the large proportion of unknown taxa, we only identified differences in relative abundance of *Cutibacterium aureobasidium* and *Streptococcus thermophilus* are considered as Atlantic salmon commensals. *C. aureobasidium* is a commensal bacterium that forms part of the bacterial flora on Atlantic salmon skin. Recent studies have shown that this bacterium can produce an in female fish antimicrobial compounds that may help protect the fish from potential pathogens^47^. Following delousing, *C. aureobasidium* displayed an immediate reduction in abundance, reaching its lowest value at 2 dpd followed by a gradual increase to 60% of its pre-delousing levels (Supplementary Fig. 2a). Interestingly, in male salmon, *C. aureobasidium* showed an opposite trend compared to females, with a rapid expansion that peaked at 2 dpd, followed by a gradual decrease at day 13, but still showing higher abundance than pre-delousing levels (Supplementary Fig. 2a). However, females show no differences in *S. thermophilus* proportion following mechanical delousing, whereas males exhibit an immediate expansion that persists even 13 dpd (Supplementary Fig. 2b). *S. thermophilus* is not commonly present in the natural bacterial flora of Atlantic salmon skin^48,49^. *S. thermophilus* is not considered to be a pathogen of fish. However, if *S. thermophilus* is found on Atlantic salmon skin, it may be an indication of environmental or mechanical stressors^50^. Further research is needed to fully understand the differences in abundance of specific ASVs between male and female Atlantic salmon following Hydrolicer treatment and their implications for the overall fish health.

Our analyses failed to identify colonization of known opportunistic or pathogenic bacterial taxa during the 13 days we surveyed the salmon skin microbial community. The consequences over a longer period post delousing and repeated delousing perturbances are unknown, and it can be speculated that opportunistic pathogens might colonize and even cause disease in individuals with imbalanced microbiome, likely in sex-dependent ways.

Our study includes several caveats. The first one is that we did not measure glucose and cortisol responses in female and male fish following delousing. Thus, it is possible that females displayed reduced stress responses to mechanical delousing, and, in turn, that translated in less pronounced changes in the skin microbiome and a quicker recovery of the community in females compared to males. This is therefore an important question that should be answered in future studies. Additionally, although the fish were overtly immature, we cannot rule out that differences in time to onset of sexual maturation may impact our results and should be taken into account in future studies. However, the fish were approximately a year from reaching sexual maturation, and thus this should not influence the results. Finally, the experimental work flow involves partial replenishment of water between the treatment of females and the treatment of males. As a result, it is possible that residual parasites from female delousing may have impacted male parasite loads.

In conclusion, our work unveils novel aspects of microbiota responses to a common fish farming practice, mechanical delousing. We report important baseline sex differences in the skin microbiome of broodstock Atlantic salmon which likely determined the differential resistance and resilience of female and male salmon skin microbial communities to mechanical delousing.

Given that microbiota resilience is considered a measure of host health^37,51^, our findings highlight that monitoring the diversity of fish microbial communities prior to any manipulations may be a useful tool to predict the time to recovery and extent of dysbiosis following such manipulation.

## Methods

### Animal history and handling

Fertilized Atlantic salmon eggs belonging to Elite Robust breeding line and hatched in March 2016 were obtained from AquaGen. After hatching the fish were maintained in the fresh water in tanks at the hatchery site Holmvåg, and all animals were vaccinated with ALPHA JECT® 6–2 (Pharmaq, Norway) in October 2016. Before transfer to sea, personnel from Aquagen sorted the fish using an ultrasound machine differentiating females and males before they were transferred to sea in their respective cages (cages 5 and 10, respectively) in May 2017. Females (72,000 individuals, average weight 98.5 g) were transferred to a cage 100 m in circumference and volume 10027 m^3^, while males (103,000 individuals, average weight 94.5 g) to a cage 120 m in circumference and volume 24717 m^3^. According to the regulation for sea lice per fish, to keep lice numbers under the required level, the fish in both cages had undergone three lice treatments using Emamectin administered orally along with feed. The lice treatments were carried out in August, October and December 2017 using doses 3.3, 5 and 10 mg respectively.

When the salmon lice treatment using Hydrolicer (SMIR, Norway) was planned in August 2018, which was 8 months since the last treatment, we planned the sampling for microbiome. Just before the treatment the average lice burden per analyzed fish was 1.05 and 0.98 for the females and males cages respectively. There were no differences between the two cages regarding detection of infectious agents, disease, or mortality. PRV was detected in both cages in June 2018. Females in cage 5 had a mean weight ± SD of 3764 ± 494 g, and males in cage 10 had a mean weight ± SD of 3690 ± 261 g both groups "intended for broodstock". We did not determine the sexual maturity of the fish in this study but based on our experience, animals should have not reached sexual maturity at this stage of the production cycle and weight. It is worth noting that at the time of sampling, fish were still approximately one year away from reaching sexual maturity and spawning stage. Water temperature was 7 ± 1 °C measured at the commencement of delousing and at the start of collecting fish for sampling on day 2. The fish were crowded by pulling together the inner net in the cage before pumping the fish into the well boat. Cage 5 containing females was deloused first at 17:20 while the males in cage 10 were deloused 7 h later. According to the general practice, the water in the well boat was only partially replenished and not treated between the treatment of fish from the two cages. However, to reduce the chances of this workflow impacting our data, skin samples were collected from male Atlantic Salmon with a time delay that exceeded the hydraulic retention time of the well boat. The general health of the fish to be deloused was good with no special health related issues or mortalities noted before or after the treatment. The mortalities in the females and males cages were 0.08% and 0.09% respectively during the week post delousing operation. The operation was considered successful with very low mortalities, or any disease outbreaks following the handling manipulation, and parasite loads were decreased to below threshold levels (albeit not quantified).

**Figure 8:**
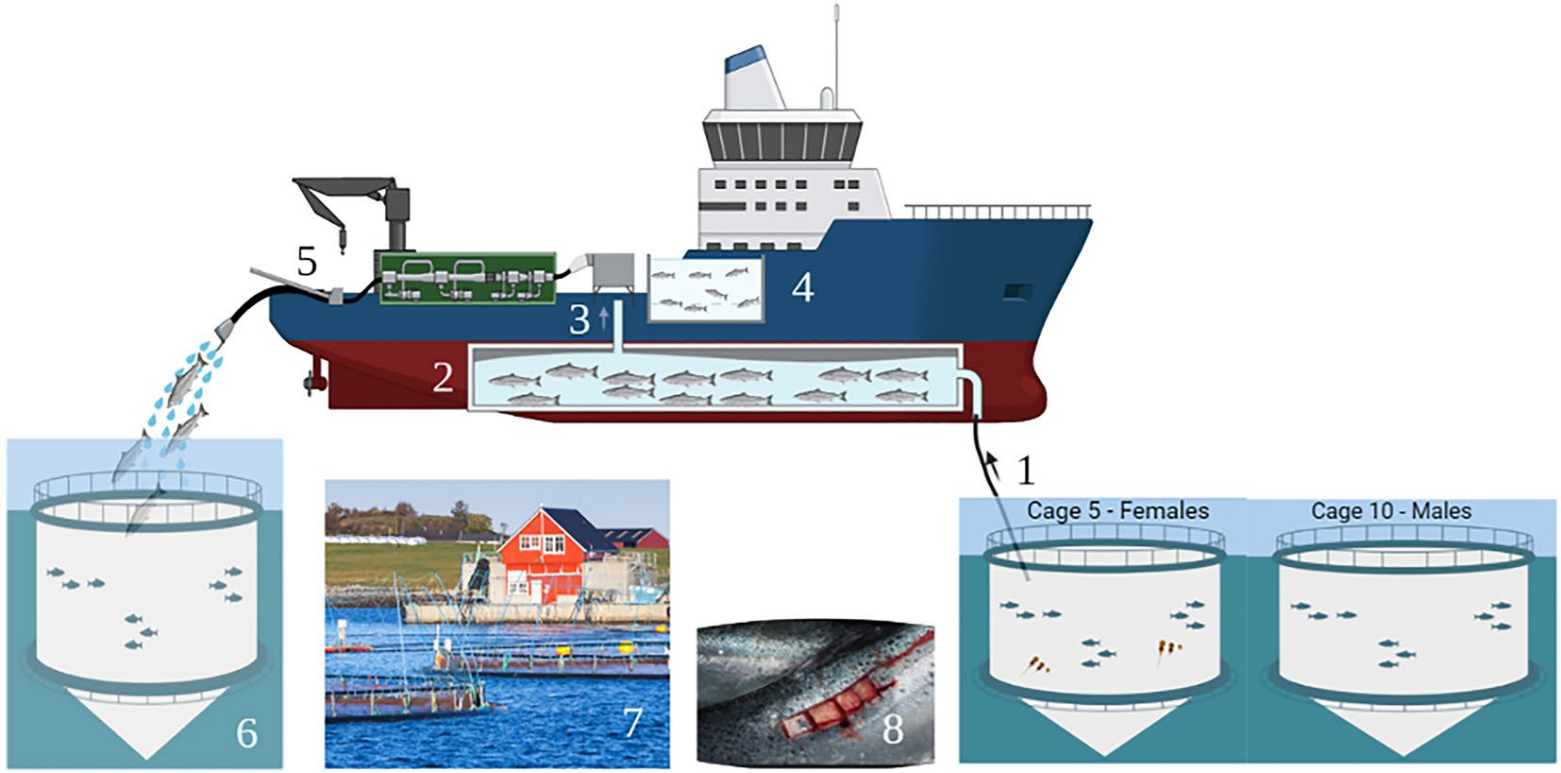
Schematic representation of the experimental set up and sampling for this study. The fish within a cage were constrained before pumping them (1) into the well boat (2) where they were allowed to settle for one hour before pumping (3) them through the Hydrolicer system. Fish exited into a collection tank (5) before they were pumped back into their respective cage (6). The fish were constrained in their respective cage before pumping them into a well boat. At time 0, day 2 and 13 post-delousing, fish were collected with a lift-up net from the cages straight into a tank (point 4). The fish collected for sampling into a tank (point 4) was transported to a land-based sampling facility (point 7), anesthetized in smaller buckets and sampled for skin from the same spot on the fish as shown in 8. Samples were collected at Time 0 before treatment, representing the fish in the cage post constraint, post treatment before they were pumped back to cages (5), and from point 6 at days 2 and 13 post treatment. Figure created with Biorender.

Figure 8 shows the overall experimental approach of the study. Briefly, female, and male salmon were sampled right before delousing treatment, and at three timepoints post Hydrolicer treatment. Fish from cage 5 were all females and fish from cage 10 were males. Cages were 90 m away from each other measured from the cage midpoint. For sampling of fish at Time 0 before handling and treatment, and at Day 2 and 13 dpd, fish were collected straight from the cage into the transport tank, and all samples were collected within 2.5 h. For delousing treatment, fish were first pumped into a well boat and were allowed to settle for one hour in the.well boat, before pumping them through the Hydrolicer. Fish to be sampled just after treatment were collected from the point where fish were being pumped out of the treatment chamber before they were pumped into their respective cage. Fish to be sampled were transferred using nets into 500 L transport tanks containing seawater and AquiS (10–12 mg isoeugenol/L seawater in the transport tank) to keep the fish calm. The tanks were transported 15 min with a boat and lifted onto land, where 5 fish/bucket were euthanized in buckets containing 30 L seawater and 20 ml Benzoak (final concentration 40 mg of Benzocaine (ethyl 4-aminobenzoate)). Fish were always handled on the right side and tail region, since sampling was performed on the left side of the fish.

The fish that were sampled were maintained in the cages as a part of the production cycle and were monitored regarding behavior changes and welfare conditions by fish health specialist at the site by the production company. The scientific team were allowed to sample the fish at the site during the routine handling procedures. Euthanasia of fish without planned experimental procedures, and further sampling from fish in production are routine procedures at a production site.

This study aimed to build knowledge about the impact of routine procedures in a production line of Atlantic salmon, here especially salmon lice treatment using Hydrolicer. The sampling procedures were carried out during the hold and normal production of salmon at the location and was not part of a designed experiment. Therefore, according to the Norwegian animal ethics board, the study did not require an application for approval from the animal ethics committee in Norway. The fish were however handled according to the ethical standards by the fish health specialist at the site.

### Sampling

Sampling of 20 fish/cage at each sampling timepoint was always carried out in the same order. First, skin samples for microbiome analysis were collected before the fish were handled further for weighing. A 0.5 cm^2^ piece of skin was collected using a sterile scalpel blade from each animal from the area below the dorsal fin and above the lateral line and placed in a 1 ml sterile sucrose lysis buffer. Fish were then placed on the sampling table with a measuring tape to note the fork length and general health observations. Samples were first placed on ice and then frozen down at − 20 °C as soon as it was possible. Samples were transported to the laboratory and stored at − 80 °C until they were used for DNA extraction.

### DNA extraction and 16S rDNA sequencing

To extract genomic DNA, Atlantic salmon skin samples (n = 15 per group) preserved in sucrose lysis buffer, were at first lysed using 2 tungsten carbide beads per tube and shaken in the Qiagen TissueLyser II for 5 min at the frequency of 30/sec. Then, 1% of CTAB reagent (hexadecyltrimethylammonium bromide, Sigma) and 1 mL of proteinase K (100 mg/ml) were added to the homogenate skin and DNA was extracted following the method previously described by Mitchell & Takacs-Vesbach 2008^52^.

Once isolated, the DNA was resuspended in 50 μL of molecular grade sterile Nuclease-free water. Skin DNA purity and concentration were measured via spectrophotometer Nanodrop ND 1000. DNA samples were diluted 1 in 10 or 1 in 20 and three independent PCR reactions were performed for each sample to amplify the V1–V3 variable regions of the prokaryotic 16S rRNA using the primers 28F 5’-GAGTTTGATCNTGGCTCAG-3’ and 519R, 5’-GTNTTACNGCGGCKGCTG-3’ (where N = any nucleotide, and K = T or G). The three PCR products obtained for each skin sample were merged and cleaned using the beads from the AxyPrep Mag PCR Clean-up Kit (Thermo Fisher Scientific) following the manufacturer’s instructions. All skin samples were then indexed with Nextera XT Index Kit v2 Set A (Illumina), where a unique combination of 2 barcodes (N and S) were ligated to the 5′ and 3′ ends of each sample. The amount of DNA in each sample was quantified using the Qubit high sensitivity dsDNA assay and normalized to a concentration of 200 ng/μl. DNA samples were then pooled together to form a library and cleaned again using the Axygen PCR clean-up kit before sequencing at the Clinical and Translational Sciences Center at the University of New Mexico Health Sciences Center. Sequencing was performed using the Illumina Miseq platform where forward and reverse paired-end sequences (300 bp in length) were amplified with the MiSeq Reagent Kit v3 (600 cycle) (Illumina).

### Microbiome sequencing analysis

Sequence data was analyzed by Quantitative Insights into Microbial Ecology 2 (Qiime2, v2022.2)^53^. Demultiplexed sequence reads were clustered into amplicon sequence variants (ASVs) using the Divisive Amplicon Denoising Algorithm (DADA2)^54^. To assign taxonomy, ASVs were aligned to the latest version of the Silva 16S rDNA database (v138)^55^. Before performing core diversity analyses, samples were rarefied to a sampling depth of 2,300 reads per sample. Then, core diversity analysis was performed considering the variables sex and treatment. Alpha diversity indices (Faith’s phylogenetic diversity and Shannon diversity) and beta diversity measures (Weighted UniFrac distances) were generated using the QIIME2 plugin. Principal coordinate analysis (PCoA) plots for beta diversity metrics were generated using the qiime2R package in RStudio version 1.3.959^56^. To predict the functional composition of sampled microbial communities, PICRUSt2 analysis was done using RStudio^57^. To better elucidate changes in microbial communities at the genus level, we performed a BLAST search on the assigned sequences for each feature ID against the NCBI database. Specifically, we selected any feature IDs with a relative frequency greater than 2 percent. Out of the 17 (ASVs) at the genus level, only three yielded blast results that allowed for their unambiguous assignment to known genera with acceptable query coverage (Supplementary Table 1).

### Statistical analyses

A mixed model ANOVA was used to evaluate the effects of sex and treatment on the skin alpha diversity metrics as previously described^58^. Differential abundance analysis was performed with the one-way ANOVA or by unpaired Student’s t-test and only differences with a *P-*value lower than 0.05 were considered statistically significant. Atlantic salmon skin 16S rRNA sequencing data were deposited under Bioproject PRJNA856342 in the NCBI Sequence Read Archive (SRA).

## Supplementary Information

Supplementary Information.

## Data Availability

All Atlantic salmon skin 16S rRNA sequencing data were deposited under Bioproject PRJNA856342 in the NCBI Sequence Read Archive (SRA).
