# Supplementary material for: Sex-dependent effects of mechanical delousing on the skin microbiome of broodstock Atlantic salmon (Salmo salar L.)
Source: Sci Rep. 2023 Jul 4;13:10824. doi: 10.1038/s41598-023-37670-4 (PMC10319866; doi:10.1038/s41598-023-37670-4)
Supplement: Supplementary file 1 — Supplementary Information. [file 41598_2023_37670_MOESM1_ESM.docx]

**Supplementary Table 1:** ASVs assigned to known genera at the genus level, with acceptable query coverage. Only feature IDs with a relative frequency greater than 2 percent were selected. Statistical significance was determined using a threshold of P<0.05, with different letters in each column indicating significantly different values.

| Species | *Cutibacterium aureobasidium* | *Staphylococcus hominis* | *Streptococcus thermophilus* |
| --- | --- | --- | --- |
| Sex-treatment | Relative abundance (%) | | |
| FD0-Pre | 4.02^a^ | 0.96^a^ | 0.27^a^ |
| FD0-Post | 2.34 ^ab^ | 2.22 ^a^ | 0.44^ab^ |
| FD2 | 0.08^b^ | 0.06 ^a^ | 0.01^a^ |
| FD13 | 2.59^ab^ | 2.41 ^a^ | 0.89^ab^ |
| MD0-Pre | 1.14^ab^ | 3.79 ^a^ | 0.00^ab^ |
| MD0-Post | 3.47^ab^ | 4.72 ^a^ | 1.13^ab^ |
| MD2 | 10.20^C^ | 3.70 ^a^ | 2.03^b^ |
| MD13 | 3.59^ab^ | 3.41 ^a^ | 1.36^ab^ |


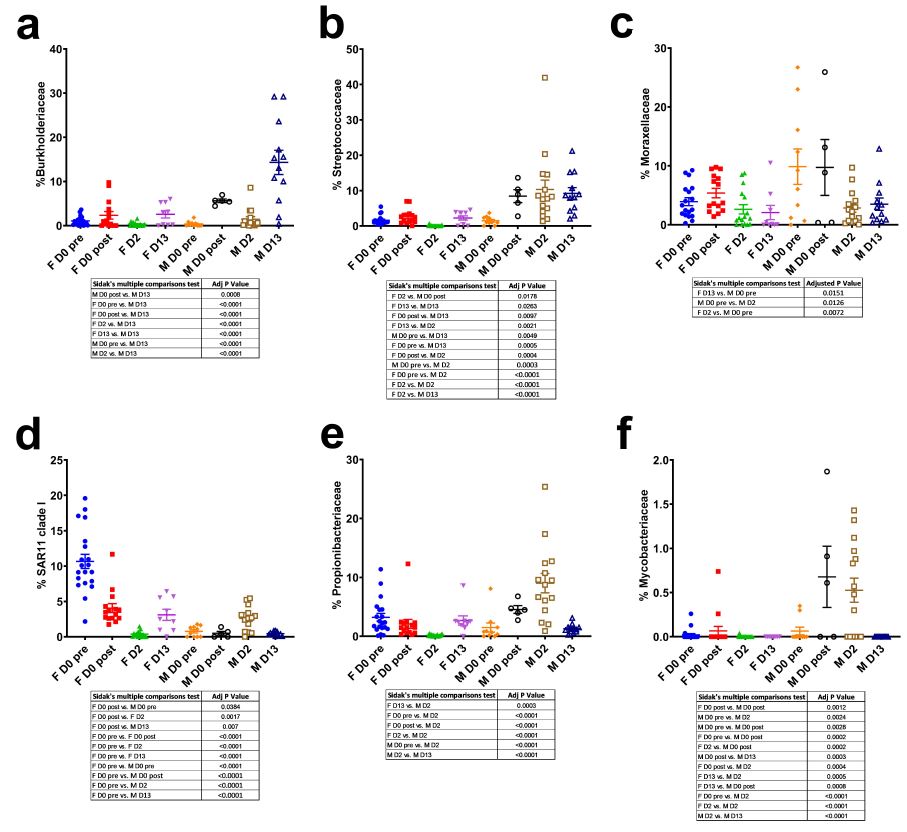


**Supplementary Figure 1**: **Delousing causes differential expansions of bacterial taxa in female and male skin microbial communities**. Relative abundance of (a) *Burkholderiaceae*, (b) *Streptococcaceae*, (c) *Moraxellaceae*, (d) *SAR11 clade I*, (e) *Propionibacteriaceae* (f) *Mycobacteriaceae* in female and male Atlantic salmon skin microbial communities at day 0 pre-delousing, day 0 post-delousing, 2 dpd and 13 dpd. *P-*values for Sidak’s multiple comparisons tests are shown in the tables underneath each graph.


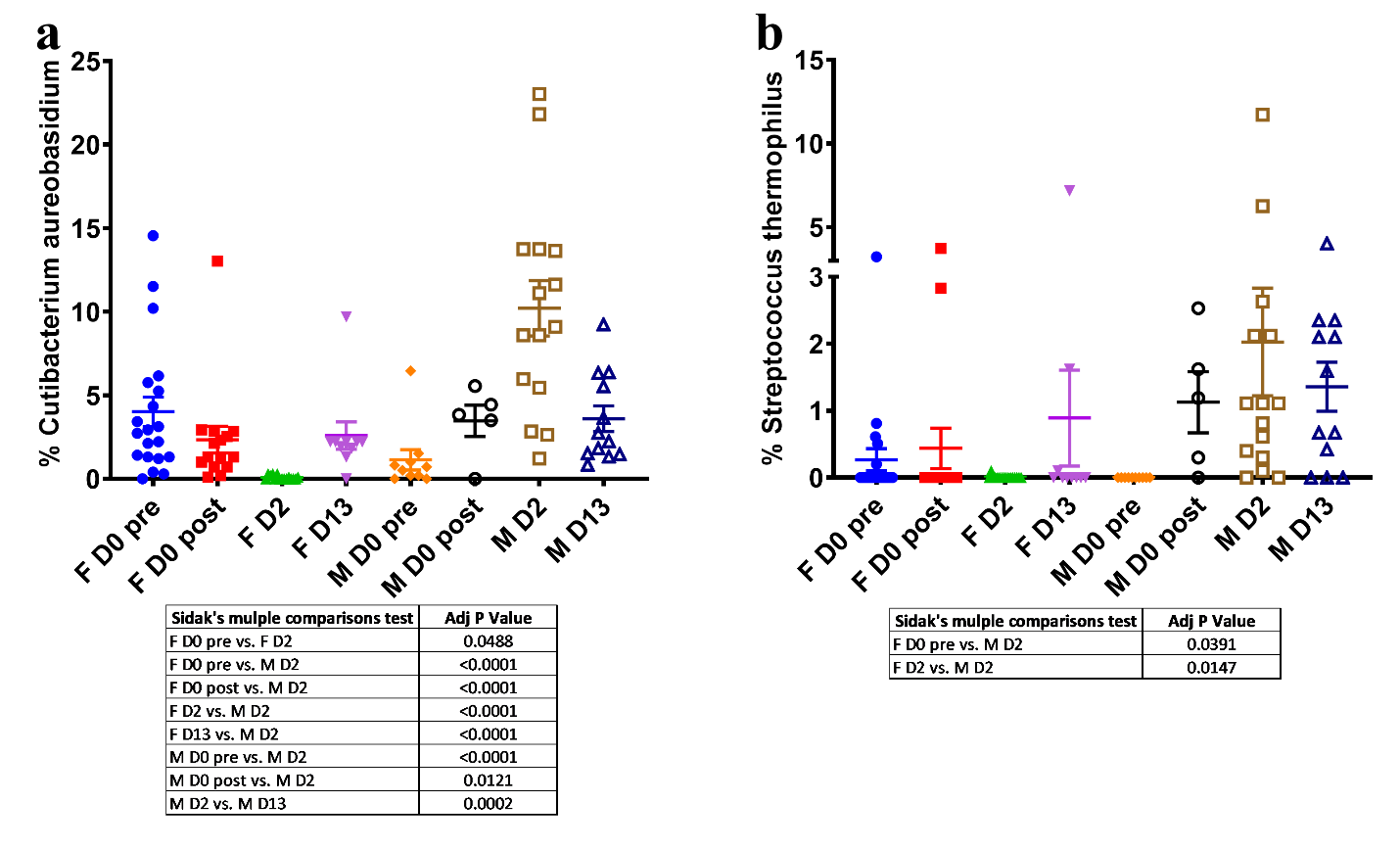


**Supplementary Figure 2:** Delousing-induced changes at genus level in Atlantic salmon skin microbial community (Only significantly different assigned ASVs with acceptable coverage.)

Relative abundance of (a) Cutibacterium aureobasidium, (b) Streptococcus thermophilus in female and male Atlantic salmon skin microbial communities at day 0 pre-delousing, day 0 post-delousing, 2 dpd and 13 dpd. P-values for Sidak’s multiple comparisons test are shown in the tables underneath each graph.
